# Platform Process for an Autonomous Production of Virus-like Particles

**DOI:** 10.1021/acsomega.4c09694

**Published:** 2025-01-21

**Authors:** Simon Baukmann, Alina Hengelbrock, Kristina Katsoutas, Jörn Stitz, Axel Schmidt, Jochen Strube

**Affiliations:** † Institute for Separation and Process Technology, Clausthal University of Technology, 38678 Clausthal-Zellerfeld, Germany; ‡ Research Group Medical Biotechnology & Bioengineering, Faculty of Applied Natural Sciences, TH Köln - University of Applied Sciences, 51368 Leverkusen, Germany

## Abstract

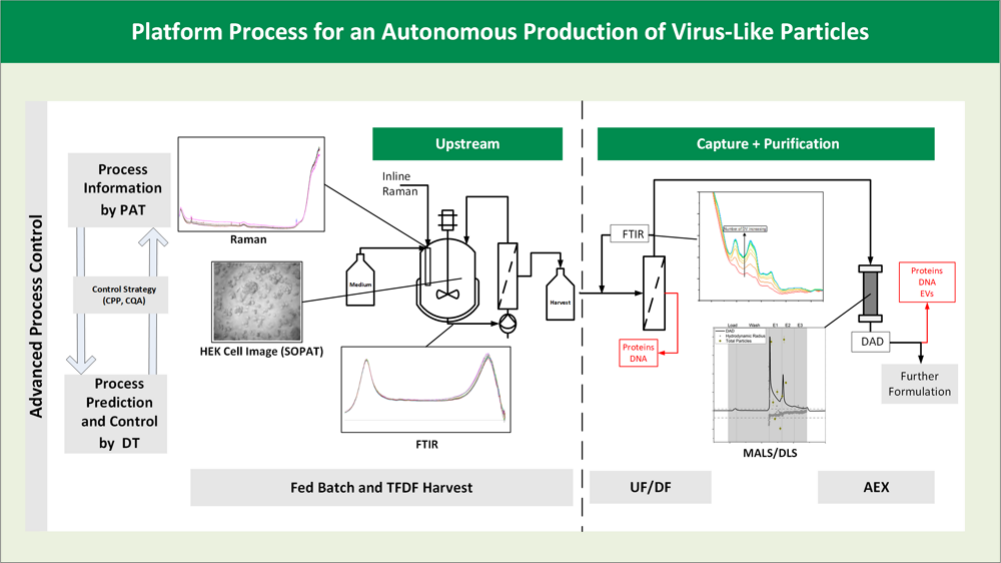

Virus-like particles (VLPs) are a promising platform as carriers for vaccination and general treatments against many pathogens. While there are long development timelines and challenges in the production of clinical-grade VLPs, this study introduces a platform process for the production and purification of those particles, aided by process analytical technology (PAT). Fed-batch cultivation and consecutive purification, including novel membrane technology and anion-exchange chromatography, showed robust process performance with design and control spaces defined in previous studies. A novel, stable HEK293F cell line generated using the highly efficient *Sleeping Beauty* transposon vector technology showed a 3.6-fold increase in productivity compared to a reference cell line established using *PiggyBac* vector components. The in situ microscope from SOPAT GmbH successfully predicted the viable cell density of a mammalian cell culture, which had been demonstrated for the first time with this type of ISM. Furthermore, Raman, FTIR, and DAD spectroscopies were able to predict the main metabolites and impurities when implemented in the upstream process. FTIR spectra also depicted changes in the buffer composition, therefore enabling robust process control for the prediction of the buffer exchange rate during diafiltration. The applied PAT strategy can deliver real-time data, which is crucial when predictive control is realized with a digital twin. Combined with a robust platform process, the stated control strategy can pave the way toward the autonomous production of VLPs.

## 1 Introduction

In recent years, the demand for effective vaccines has increased steadily as they provide an effective treatment against various pathogens. Virus-like particles (VLPs) as a carrier platform promise vaccination against many pathogens and diseases.^1^ They mimic real viruses and can be more effective than conventional vaccines due to their enhanced immunogenicity caused by a highly repetitive, antigen-presenting structure.^2^ As they do not carry genomic material, VLPs are safe to handle and can be used as a nanocarrier for the delivery of immunogens, proteins, and DNA.^2^

Gag-VLPs represent the structure of human immunodeficiency virus (HIV), and the immature particles consist of core Gag and envelope Env proteins. They are currently under investigation as a vaccine candidate against HIV-1 and as a framework for presenting various other antigens with the potential to become a platform for flexible vaccine production.^3^ To overcome the hurdle of long timelines, platform processes can shorten the development of suitable processes significantly and also allow continuous biomanufacturing.^4^ Clinical-grade production of these particles is still challenging and demands robust unit operations with design spaces that fit the needs in the quality-by-design (QbD) context.^3,5^

**Figure 1:**
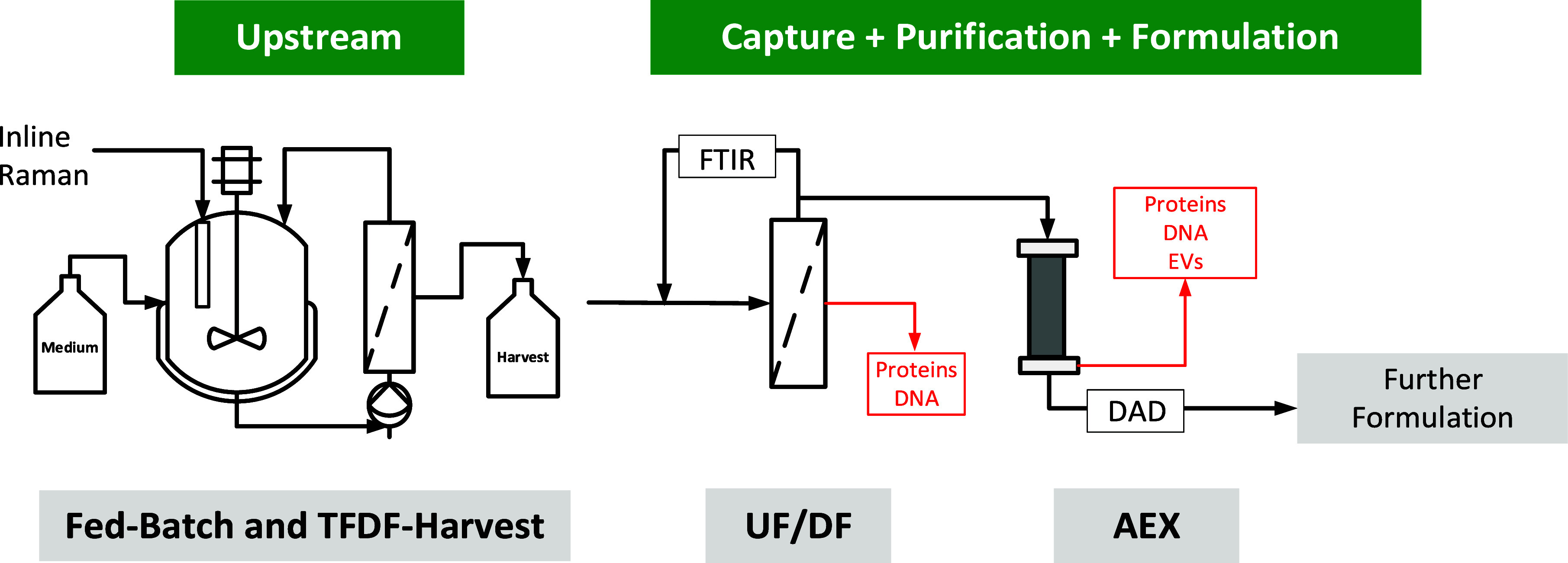
Overview of the HI-VLP batch production process for harvesting and downstream processing.

In a recent paper, we suggested design spaces and principles to control the continuous production of VLPs with a digital twin (DT) verified by simulation studies.^6^ A process consisting of a fed-batch/continuous production of VLPs in mammalian HEK293 cells with TFDF harvest followed by a batchwise downstream process (DSP) with a UF/DF step, an anion-exchange chromatography (AEX), and a final UF/DF step for formulation (see Figure 1) showed a 2-fold productivity gain compared to a standard process and an additional 20% at 99.9% reliability when controlled by a DT. To achieve a functional, autonomous process, a high rate of real-time data is needed that can be realized by process analytical technology (PAT). Spectroscopic sensors such as Raman, FTIR, and DAD are favored in PAT as they offer accurate measurements of many metabolites and impurities, often defined as critical quality attributes in the QbD design process.^7,8^ These sensors have a short delay between measurement and evaluation and thereby can continuously feed a DT. Evaluation of spectra is mostly done by chemometric calculations like principal component analysis.^7^

Imaging techniques such as in situ microscopy (ISM) have gained recent interest for gas–liquid and liquid–liquid systems as well as for bioprocesses as they offer direct insights into morphological changes in a process.^9,10^ For bioprocesses, they were successfully used to monitor cell density and changes in morphology to ensure optimal growth conditions by adapting critical process parameters (CPPs) like pH, temperature, or shear stress.^11^ Evaluation of image parameters (e.g., gray value) is preferably automated via image analysis software. Advanced algorithms like neuronal networks are also able to quantify morphological changes when trained beforehand. With the ability to generate real-time measurements, ISM provides a powerful PAT tool to feed DTs within an autonomous process.

The purpose of this work was to examine the performance of the stated platform process in successfully producing and purifying Gag-VLPs when different stable HEK293F cell lines were used in the upstream process. These lines differ only in the transposon system used for stable transfection, which should lead to higher productivities and VLP titers. Here, we investigated three lines (PBp, PB, and SB) prepared by the Stitz Lab with two different transposon vector systems applied: *PiggyBac* (PB) and *Sleeping Beauty* (SB).^12−14^ PB-based cell lines were either established with a plasmid vector (PBp) or an mRNA vector (PB) to mediate the integration of Gag and Env encoding expression cassettes into the cells’ genomes, whereas for SB, only the mRNA vector was used.^15^

Raman, FTIR, and DAD sensors were investigated regarding their performance to accurately measure metabolites and impurities during cultivation. For the first time, the in situ microscope from SOPAT GmbH was used to examine its potential for monitoring the viable cell density (VCD) in a mammalian cell culture. These insights can accelerate the decision-making process for VLP development and introduce new methodologies for the application of PAT with the goal of an autonomous process.

## 2 Materials and Methods

### 2.1 Cultivation of HEK293 Cells

The recombinant cell lines investigated in this study were transfected by transposon vector systems consisting of the HEK293FMos1.Gag/Mos2S.Env vector (plasmid (p) or mRNA encoding for the respective transposases) and derived from either PB or SB transposons. The vector encodes for Gag proteins, which are composed of mosaic epitopes derived from different HIV-1 variants. In addition, the transfected cell lines coexpress mosaic envelope proteins.^12^ The main difference between those stable cell lines is suggested by the transposition efficiency of the SB construct, which should therefore lead to higher productivity and VLP titer. A comparison study was conducted to determine the potential of SB based on data published in a previous study.^6^ Cells were cultivated in a Gibco Dynamis medium (Thermo Fischer Scientific, Waltham, USA). The seeding cell concentrations varied between 0.5 and 1.5 mio. cells/mL, and cell concentrations were determined daily using the Trypan Blue exclusion method with a CEDEX XS system (Roche Holding, Basel, Switzerland) for automatic cell counting. Glucose and lactate concentrations were determined every day from clarified cell culture samples by enzymatic-amperometric measurement in a LaboTRACE Compact (TRACE Analytics GmbH, Braunschweig, Germany).^16−18^

Cultivations were performed in fed-batch, and feeding was started after 3–4 days when the glucose concentration had reached <2.0 g/L. The culture was fed with HEK FS 2 feed (Sartorius AG, Göttingen, Germany) to 2.5–3.0 g/L.

Cell lines were compared on the basis of the space-time yield (STY). The total number of VLPs (*N*_total_) is calculated from the determined product concentration (*c*_STR,t_) and the culture volume (*V*_STR_).^16^
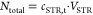
1

The STY describes the amount of VLPs produced in relation to the reactor volume and the overall process duration (*t*_total_).^16^
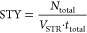
2

### 2.2 Harvest of VLPs via TFDF in the TFF Mode

For harvesting of the fed-batch, the same process conditions were applied as for the PB process. A membrane pump was used (QuattroFlow, Quattroflow Fluid Systems GmbH & Co. KG, Hardegsen, Germany) in order to reduce the shear forces acting on the cells and the product. The flow rate for the TFF process was set to 1.6 L/min,^19,20^ resulting in a shear rate of 2459 s^–1^.^17^ The TMP was manually increased to a maximum of 0.3 bar via a valve on the retentate side when the LMH decreased. A 30 cm^2^ TFDF-30 module (Repligen, Waltham, Massachusetts, USA) with a 2–5 μm cutoff was used to clarify the harvest. The process was divided into three sections. First, a concentration by a factor of 1.6 was performed, followed by washing with a 0.6 diafiltration volume culture medium and finally another concentration by a factor of 1.6.^19^

#### 2.2.1 Determination of Blocking Mechanism

In the TFF mode, separation of particles due to the cross-flow must be considered.^21−23^ Based on the single physical equation derived by Hermia, which enables the establishment of a connection between the four blocking mechanisms, the latter was determined for TFF using the index *n*. The values correspond to *n* = 2 for complete blocking, *n* = 1.5 for pore filling/standard, *n* = 1 for intermediate, and *n* = 0 for cake formation. Additionally, the consideration of cross-flow removal from the membrane surface was incorporated into the analysis.

### 2.3 Ultra- and diafiltration

The initial product purification and concentration for the subsequent chromatography step were performed with a Sartorius SARTOFLOW Slice 200 benchtop system (Sartorius, Göttingen, Germany). A hollow fiber module with a cutoff of 300 kDa (Explorer12 ReUse 0.5 mm, Sartorius AG, Göttingen, Germany) was used. The starting medium was the conditioned medium harvested at the end of the fed-batch cultures. After a concentration by a factor of 3, a buffer exchange was performed with seven diafiltration volumes, corresponding to a residual salt content of 0.8%, to MPA wAEX buffer (weak AEX). The experiment was performed at a transmembrane pressure of 0.5 bar and a shear rate of 3738 s^–1^.^17^

### 2.4 Anion-Exchange Chromatography

The UF/DF retentate pool (AEX feed) was loaded onto a weak anion exchanger (5 mL, Poros GoPure D50, Thermo Fisher Scientific Inc., Waltham, MA, USA) without further sample preparation. Buffer A (MPA) was 50 mM phosphate buffer with 5% sucrose and 2 mM magnesium chloride at pH 6. The elution buffer (MPB) contained additionally 1 M NaCl.^24−27^

Equilibration was performed with 10 column volumes (CVs) of MPA. The AEX feed was then loaded onto the column. Elution was performed in three steps. The first was at 20% MPB to elute initial impurities such as proteins, and the second was at 75% MPB to elute the product. Each step was held for 2 CVs. Finally, the remainder was eluted with 2 CV 100% MPB^24−27^ (5 CVs for PB runs). The flow rate was set at 0.26 CV/min.^24^ After elution, the column was equilibrated again with MPA for 10 CVs, followed by subsequent sanitization with 1 M NaOH for 10 CVs. For fractionation, UV absorbance was observed at 280 and 260 nm as supplementary wavelength.

### 2.5 Analytical Methods

#### 2.5.1 p24-ELISA

The p24 enzyme-linked immunosorbent assay (p24-ELISA) was used for quantification of VLPs. The VPK-107-H HIV p24-ELISA (Bio-Cat GmbH, Heidelberg, Germany) was performed according to the manufacturer’s instructions and analyzed at 450 nm with a multiplate reader (TriStar^2^, Berthold Technologies GmbH & Co. KG, Bad Wildbad, Germany). For the conversion of the resulting concentration into the actual VLP concentration, a number of correction factors and additional calculations were introduced as stated in Gutiérrez-Granados.^6,28^ The first correction by a factor of 10 is done to make up for the underestimation of immature p55 particles that are produced by the cell lines instead of p24. Also, a second factor of 2.3 was implemented to correct for the higher molecular weight of p55 compared to p24.

#### 2.5.2 PicoGreen dsDNA Detection

The DNA concentration is quantified using the Quant-iTTM PicoGreen dsDNA Reagent kit (Thermo Fisher Scientific, Waltham, USA) and performed according to the manufacturer’s instructions. The samples, diluted if the concentration exceeds the calibration range, are determined in duplicate at an excitation wavelength of 480 nm and a measured emission wavelength of 520 nm.

#### 2.5.3 Bradford Assay

The Pierce Bradford Protein Assay kit (Thermo Fisher Scientific, Waltham, USA) is used to determine the total protein concentration and is carried out according to the manufacturer’s instructions using the microwell method. All samples are determined in duplicate and diluted if necessary. The evaluation is carried out by means of an absorbance measurement at 595 nm. Interferences due to high levels of glucose, for example, have to be taken into account when evaluating the assay.

#### 2.5.4 Nanoparticle Tracking Analysis

The particle concentration of the VLP samples is measured by nanoparticle tracking analysis (NTA) using the ZetaView 30× (Particle Metrix GmbH, Ammersee, Germany) at a wavelength of 520 nm and calibrated with a 100 nm polystyrene standard. Cultivation samples were centrifuged at 100 × *g* for 3 min beforehand to separate the pellet and the supernatant. The pellet was resuspended in a fresh culture medium. Each sample was diluted with water to maintain the measurement range of 1E7 to 1E8 particles·mL^–1^. The number determination is carried out over 5 cycles with a total image acquisition time of 60 s. Three recordings are made per sample.

#### 2.5.5 Dynamic Light Scattering

The particle sizes were determined by using dynamic light scattering (DLS) measurements. The Malvern Zetasizer Nano ZS ZEN3600 (Malvern Panalytical Ltd., Malvern, UK) was used, and triplicate measurements were carried out at room temperature. The intensity distributions and mean diameters of reproduced measurements were averaged. Measurements that were not comparable, e.g., due to particle sedimentation or poor mixing of the sample, were excluded. As in the case of NTA, both the supernatant and the pellet were measured.

#### 2.5.6 Amino Acid Analytics

Amino acid concentration was measured by RP chromatography (InfinityLab Poroshell 120 HPH-C18; 3.0 × 100 mm; 2.7 μm; Agilent Technologies, Santa Clara, USA) and precolumn derivatization of amino acids with an orthophthalaldehyde (OPA) reagent in a basic medium. Borate buffer was used as mobile phase A and 70% (v/v) acetonitrile/water for elution via a gradient (0–100% for 20 min at 0.64 mL/min flow rate). Samples were also diluted (1:5) and measured to ensure complete derivatization. The column was tempered to 40 °C for better separation.

#### 2.5.7 Process Analytical Technology

For the evaluation of suitable sensors for the measurement of process parameters, as well as metabolite and impurity profiles, FTIR ReacIR 702L (Mettler Toledo Inc., Columbus, Ohio, USA), DAD SmartLine UV Detector 2600 (Knauer GmbH, Berlin, Germany), and SOPAT MA achromatic endoscope (SOPAT GmbH, Berlin, Germany) were used offline to generate respective data and spectra from daily cultivation samples. Raman spectra generated with RamanRxn2 (Endress+Hauser GmbH+Co.KG, Rheinach, Switzerland) were measured inline during the cultivation as this probe was autoclavable without further adaptions. Representative samples were obtained from the reactor and directly measured offline with the respective probe. The homogeneity of the sample was ensured by gentle mixing.

The Raman probe was calibrated, and verification was done with 70% 2-propanol according to the manufacturer’s instructions before the start of the cultivation. Three measurements with an integration time of 30 s were taken every 15 min over the course of cultivation.

Three FTIR measurements were taken, each consisting of 32 individual spectra. When applied during UF/DF, a flow cell was used instead of a sampling probe and placed at-line supplied via a LaPrep P130 HPLC pump (VWR International GmbH, Radnor, PA, USA) with a flow rate of 2 mL/min. The flow cell was cleaned beforehand, and the validity of the setup was determined as mentioned above. For the DAD flow cell, samples were also supplied by the HPLC pump with the same flow rate. Before each measurement, the flow cell was flushed with water until a constant signal was reached. Afterward, the flow cell was flushed with water, cleaned with 0.1 M NaOH, and stored in water or 20% EtOH.

The spectra of the three probes were analyzed with an Unscrambler V12.2 (Aspen Technology Inc., Bedford, MA, USA). For each set of spectra, a second-degree polynomial Savitzky–Golay transformation was performed with different numbers of smoothing points for the elimination of additive and noise effects and a further SNV transformation for the elimination of multiplicative effects, if appropriate.^17^ Fingerprint regions were identified, and regression models were established between cultivation data and spectra by PLS. For analysis, roughly two-thirds of the obtained data were used for model training, while one-third was used as a validation data set. Calculation of the partial least-squares regression (PLSR) model was done by using the NIPALS algorithm. A detailed description of the application of PLSR can be found in Esbensen et al.^29^

The SOPAT probe (MA probe with U-light) was directly introduced into the sample. For each sample, 80 images were taken with a gain of 0%, strobe intensity of 1, and an exposure time of 48 μs. Part of the images was utilized for model calibration within the SOPAT “Concentration-Calibration” (CoCa) tool to find features that represent the course of VCD data generated by CEDEX measurements. Features with a good fit were used for further verification of the remaining image sets. Visualization of the results was done in the SOPAT Result Analyzer.

## 3 Results and Discussion

### 3.1 Cultivation of HEK293 Cells

**Figure 2:**
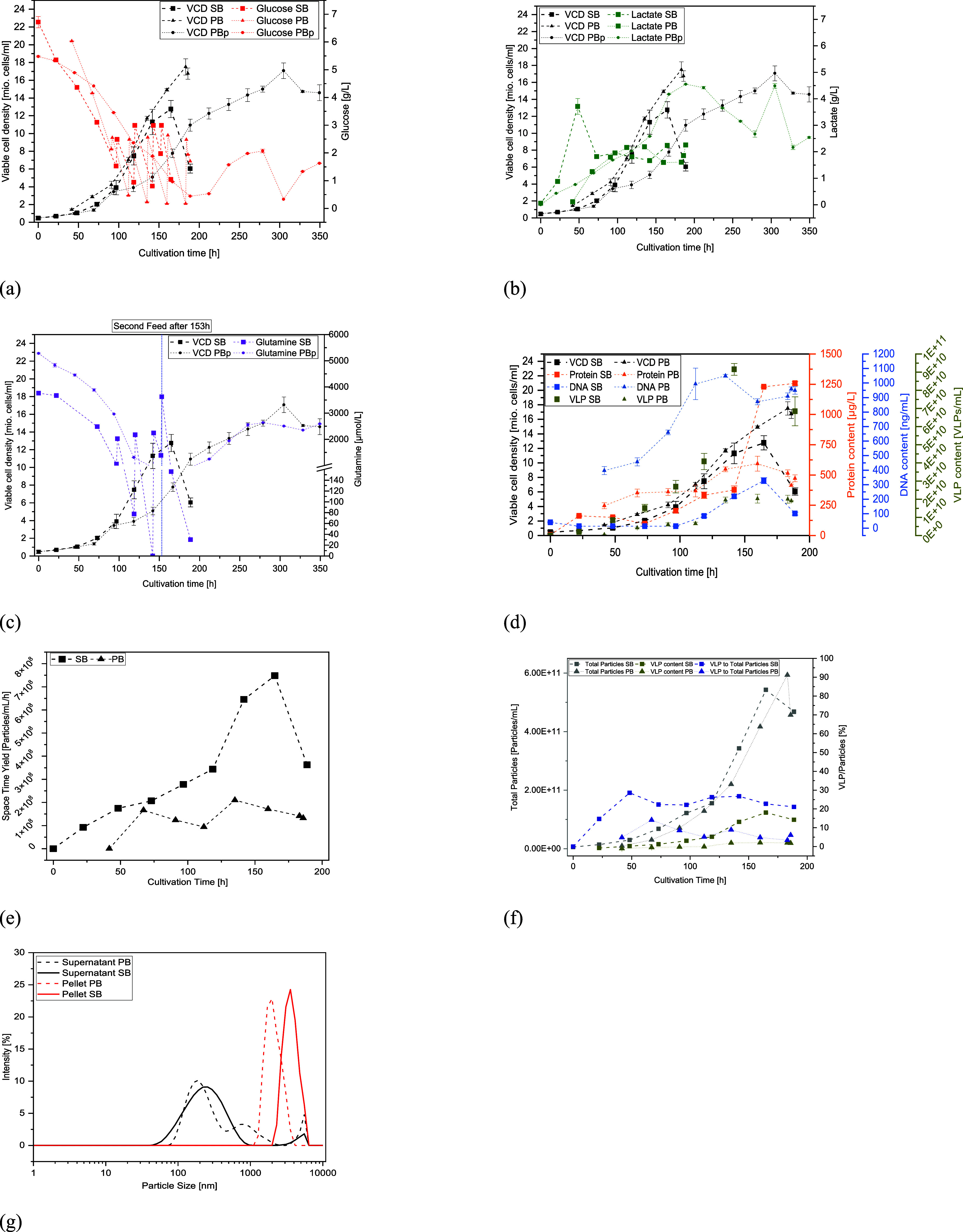
Analytical data and comparison between SB- and PB-based cell cultivation. (a) Glucose and (b) lactate data, (c) total particle content by NTA compared to p24-ELISA, (d) DNA and total protein impurity as well as product analysis with p24-ELISA, (e) overall STY, (f) glutamine data, and (g) DLS analytics from cultivation broth before harvest.

All cultivations were performed in the fed-batch mode. Glucose was kept between 2.5 and 3.0 g/L based on daily consumption rates to prevent any limitations of the primary carbon source. As can be seen in Figure 2a,b, growth rates and general metabolite profile were comparable for SB and PB until ∼140 h. The growth rate for PBp was much slower, and therefore, a prolonged cultivation compared to PB and SB was needed to reach comparable VCD values. While glucose and glutamine profiles for PBp were comparable, lactate values reached higher concentrations during cultivation with a maximum of 4.5 g/L compared to 2.6 g/L for PB and 2.2 g/L for SB. Higher lactate concentrations are caused by cell stress, which could be responsible for the lower growth rate observed in the PBp cultivation. For SB, the metabolites were not only measured daily but also evaluated directly afterward to outrule substrate limitations. As glutamine limitations were expected between two feeding points, a second feed on the same day (∼153 h) was implemented with the HEK FS2 medium to prevent glutamine limitations (Figure 2c). Afterward, the growth rate for SB decreased, and the lactate values rose with no sign of recovery to the initial growth rate, whereby a strong cell death could be observed, with a consequent termination of the cultivation. For the PB and PBp cultivation, no additional feed was implemented, and the cultivation was stopped when a decrease in the VCD was observed. Because the differences between PB and PBp were small in terms of impurity profile, general metabolism, and the reason that both lines were transfected with the same transposon, only PB was compared to SB for the remainder of the study based on the assumption that the PB and PBp process will behave in a comparable manner.

Impurity profiles (Figure 2d) differed between both cell lines as DNA concentrations were generally higher for SB. The protein concentrations remained comparable until ∼153 h, where the protein content for SB increased. Despite a lower VCD, the overall product titer of VLPs obtained from p24-ELISA (Figure 2d) was 3.6 times higher for SB as were the STYs (Figure 2e) compared to the PB cultivation.

Analyses of particles with NTA (Figure 2f) revealed comparable total particle concentrations between SB and PB. The ratio of VLPs to total particles steadily decreased over the course of the cultivation, with around 20% for SB and 6% for PB. DLS analysis of particle size distribution (Figure 2g) of the unconditioned harvest showed comparable distributions in the pellet (1–8 μm) as well in the supernatant (50–1100 nm).

### 3.2 Evaluation of PAT Sensors during Cultivation

**Figure 3:**
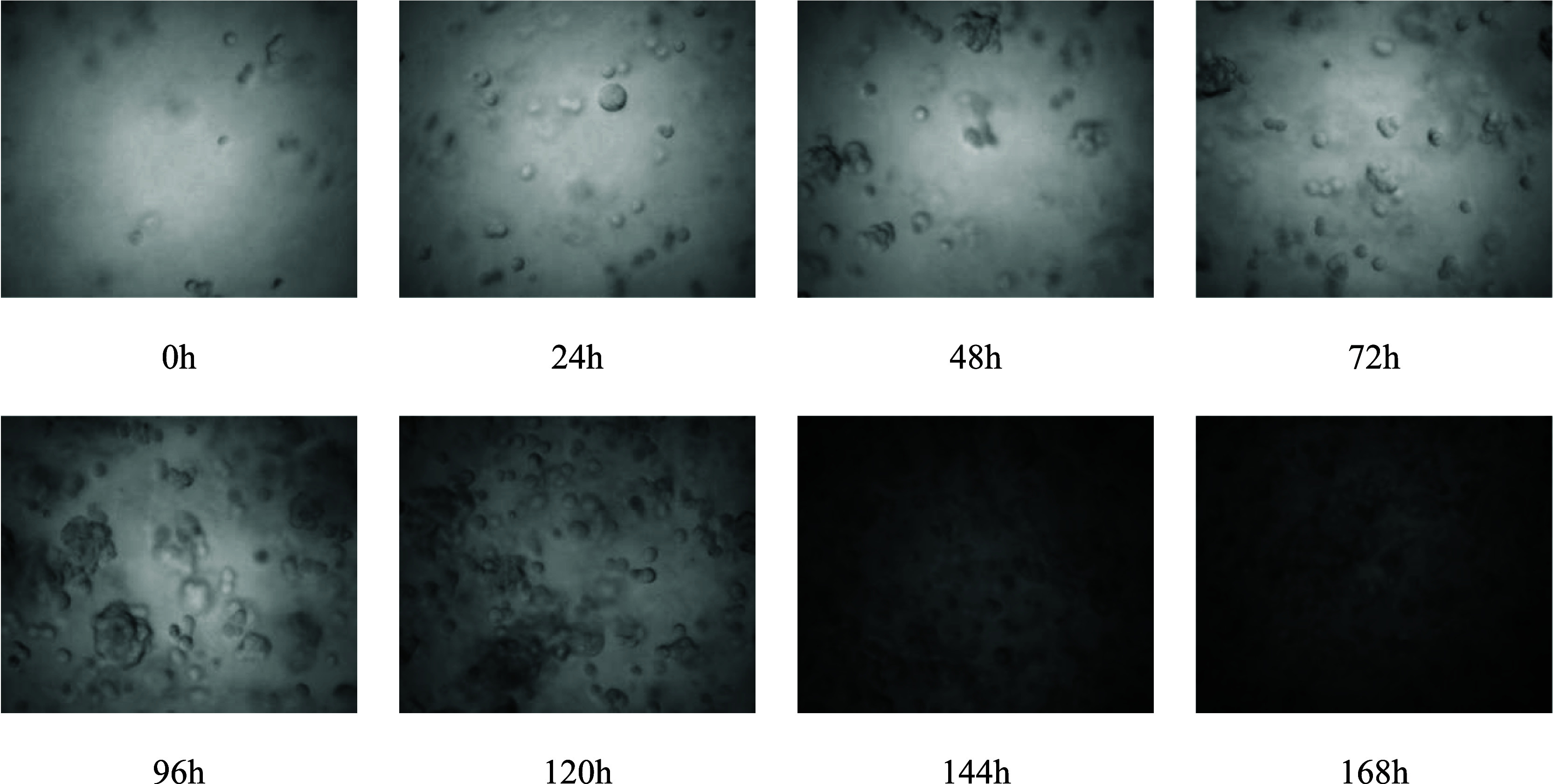
SOPAT images taken at the given time points over the course of fed-batch cultivation with SB. Darker images indicate an increase in overall cell density and other particles (e.g., cell debris).

The samples taken during cultivation were examined using the SOPAT MA probe (resolution: 1.5–250 μm) to enable online monitoring of the HEK293 cells and their aggregates. With increasing cultivation time, a change in the gray value (the images are getting darker) and the particle shape can be observed due to the increase in the number of cells and particles (Figure 3). Although cell structures were not clearly visible in the later stage of cultivation, these images were still analyzed as the entirety of changes (e.g., gray value) within an image was evaluated. Images were analyzed with the SOPAT CoCa tool. The CoCa tool consists of a total of 15 so-called “features”, whereby each feature consists of mathematical operators to describe an image. These features were evaluated against the VCD over the course of cultivation in order to identify correlations. As the death phase comprises only one data point, this was excluded from the description of cell growth.

Images from days 0, 2, 3, 5, and 7 were used for calibrating the model, and the remaining three data sets were applied for model validation (days 1, 4, and 6). The calibration against feature 7 from analysis with the CoCa tool exhibited the best results with a correlation coefficient of *R*^2^ = 0.992. For verification, the errors for the predicted VCD values were between 3 and 10%, with higher deviations for lower cell densities, which are significantly lower than the determination of the cell number using the Trypan Blue method with the CEDEX cell counter. Typically, this is between 8 and 15% for the latter method, whereby the error is higher (20–38%), particularly at lower concentrations, such as at the start of cultivation, and at high concentrations due to dilution. As the verification of the calibrated SOPAT model shows, the viable cell concentration can be measured very reliably using the MA probe. By using this in a running cultivation, the VCD can be monitored online without having to take a sample. In addition, because it is an image-based method, aggregation and any morphological changes can be observed. To quantify a culture's morphology, machine learning with advanced algorithms should be implemented, which then could provide information about the cell's condition (e.g., aggregation rate) and ultimately be used to adapt certain process parameters instantly within an autonomous process.^11,30^

For automation in the context of a QbD process, it is essential that information about the CQAs and CPPs is available in real time so that appropriate process control, ideally via DTs, can take place and consistent product quality can be guaranteed. For this purpose, in addition to the SOPAT probe, Raman, FTIR, and DAD spectra were recorded, statistically evaluated, and analyzed with regard to their predictive accuracy of glucose, protein, DNA, viable cell, and product concentration. The raw spectra were plotted to identify the general differences over the course of cultivation. After reducing the spectra to the fingerprint region, descriptive statistics, which represent the spectra over the mean of all spectra to describe scatter effects, can be used to select specific transformation methods, e.g., to eliminate additive or multiplicative effects.^29^ Two-thirds of the data were then used to create a PLS (NIPALS) model, and one-third of the data was used to verify the model. The model performance achieved, which is evaluated using the *R*^2^ and the RMSE value, for all PAT detectors and target values is shown in Table 1.

**Table 1:** Correlation Coefficient and Root-Mean-Square Error (RMSE) Values of Training (train) and Validation (val) Sets for the Applied PLS Model

sensor	parameter	glucose(train, val)	protein(train, val)	DNA(train, val)	VLPs(train, val)	VCD(train, val)
Raman	*R*^2^	0.99, 0.93				
	RMSE	0.15, 0.51 (g/L)				
FTIR	*R*^2^	0.98, 0.88	0.96, 0.97		0.94, 0.89	
	RMSE	0.32, 0.62 (g/L)	85, 69 (μg/mL)		6.9 × 10^8^, 1.1 × 10^9^ (VLPs/mL)	
DAD	*R*^2^		0.94, 0.91	0.96, 0.94	0.98, 0.81	0.94, 0.85
	RMSE		82, 133 (μg/mL)	17, 17 (ng/mL)	3.0 × 10^8^, 1.4 × 10^9^ (VLPs/mL)	0.79, 1.88 (Mio. Cells/mL)

As expected, the detection and prediction of the glucose concentration as a critical substrate works very well with both Raman and FTIR. With an *R*^2^ of 0.99 in training and 0.93 in validation as well as lower RMSE values of 0.15 g/L (training) and 0.51 g/L (validation), Raman performs slightly better for the system under investigation compared to FTIR (*R*^2^ of 0.98 (training) and 0.88 (validation); RMSE of 0.32 g/L (training) and 0.62 g/L (validation)). The total protein concentration, which is one of the main impurities, can also be described well with FTIR (*R*^2^ of 0.96 (training) and 0.97 (validation); RMSE of 85 μg/mL (training) and 69 μg/mL (validation)). Since proteins are known to have an absorption maximum at 280 nm, the description with a DAD detector is also possible and is only slightly less accurate than with FTIR with *R*^2^ values of 0.94 in training and 0.91 in validation as well as RMSE values of 82 μg/mL (training) and 133 μg/mL (validation).

In the raw spectra of the DAD, a supersaturation at high cell numbers (approximately 10 million cells/mL) can be seen, which could be an explanation for the somewhat less precise model. Nevertheless, only the DAD detector achieved a sufficiently good model to predict the low concentrated (in the ng/mL range) DNA (*R*^2^ of 0.96 (training) and 0.94 (validation); RMSE of 17 ng/mL (training and validation)).

A DAD detector is also suitable for predicting the VCD (*R*^2^ values of 0.94 (training) and 0.85 (validation)). Due to the observed supersaturation and the high RMSE value in the validation (1.88 million cells/mL), the tracking of the VCD via SOPAT is to be favored. In addition, aggregation and morphology can be observed with SOPAT. In order to evaluate these parameters automatically, further processing methods and AI image analyses are necessary.

Both DAD and FTIR can achieve good results for determining the product concentration. With an *R*^2^ of 0.98 and an RMSE value of 3.0E8 VLPs/mL, the DAD training model reproduces the actual concentrations slightly better than the FTIR (*R*^2^: 0.94; RMSE: 6.9E8 VLPs/mL). In contrast, validation with the trained FTIR model is slightly better. Since the RMSE values for both are in the range of the typical analytical error using ELISA, both detectors are suitable for the detection of the VLP content. The results indicate Raman spectroscopy as a recommended method for monitoring glucose levels and DAD as a complementary method for monitoring impurities and the VLP titer.

### 3.3 Harvest via the TFDF Filter in the TFF Mode

The SB fed-batch was also harvested using a TFDF filter module in the TFF mode under the same process conditions as stated in the previous study with PB.^6^ A high feed flow rate of 1.6 L/min was achieved by using a diaphragm pump to achieve effective particle removal on the retentate side of the filter module. TMP and therefore LMH were controlled via a retentate valve. Initially, the valve was completely opened and successively closed when the initial LMH decreased to a maximum TMP of 0.2 bar.^19,20^ Higher TMPs force particles deeper into the depth filter layer, leading to faster filter fouling, which should be avoided^31^

Cell culture harvest was processed by removing the dead volume of the system via the permeate following the first concentration step by a factor of 1.6. A wash step with culture medium was applied (DV 0.6) followed by a second concentration step (CF 1.6). The total amount of permeate generated by this unit operation therefore equaled the working volume at the end of the fed-batch cultivation, with a lower working volume for PB*.*

**Figure 4:**
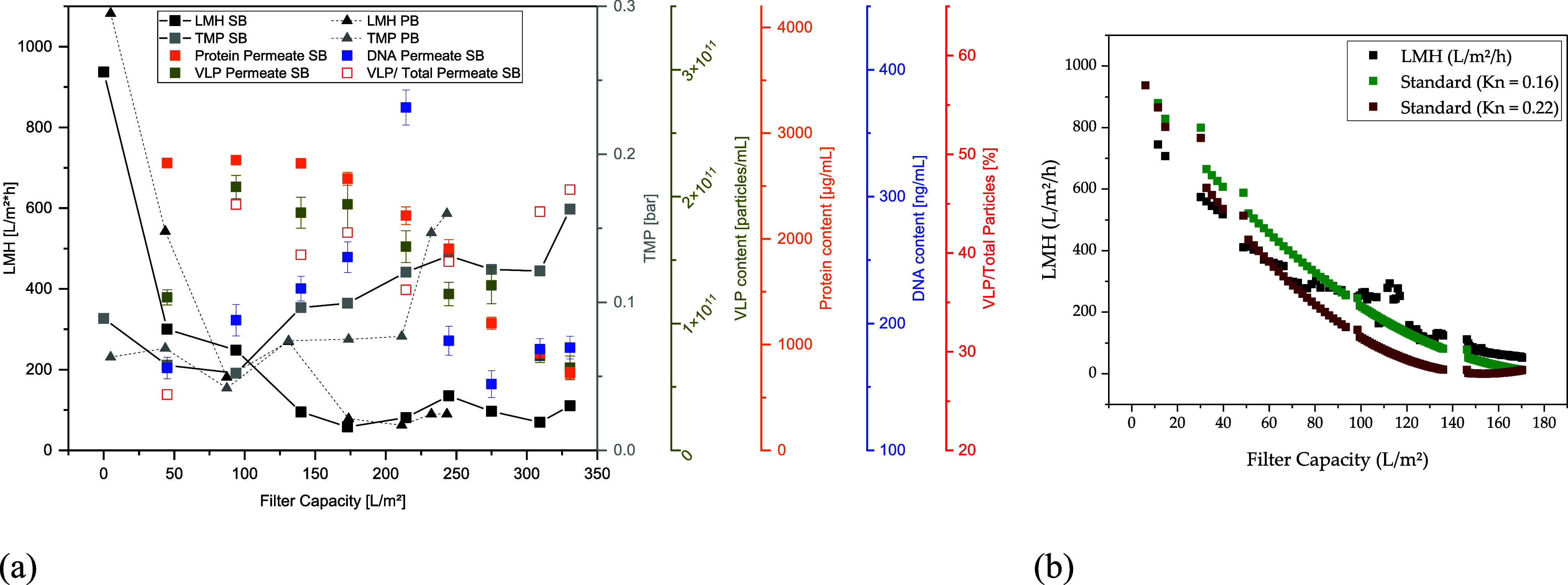
Process and impurity data of the TFDF process. (a) Comparison of permeate flux and TMP between SB- and PB-based cells as well as analytical data for the SB run. (b) LMH prediction for the SB run based on the standard blocking constant estimated from the PB run (red) and the SB run (green).

Before harvesting both the PB*-* and SB-based cultures, the supernatant of the bioreactor sample mainly contains particles in the range <400 nm, whereas the pellet consists mainly of particles >1 μm (Figure 2g). The sum of intensities of particles in the cutoff range of 2–5 μm is 30% lower for the PB cell line, and the supernatant showed a second peak consisting of particles in the range of 500–1500 nm. This may have caused the initially comparable LMHs of 1082 L/m^2^/h (PB) and 956 L/m^2^/h (SB) to drop slightly faster to 43.6 L/m^2^/h in the PB-based cultivation compared to 51.6 L/m^2^/h in the SB-based cultivation during the concentration step as smaller particles like cell debris tend to accumulate in the depth filter layer of the TFDF membrane over the course of filtration^32^ (Figure 4a). However, overall process performances between both runs were comparable, with similar courses for permeate flux and TMP.

**Figure 5:**
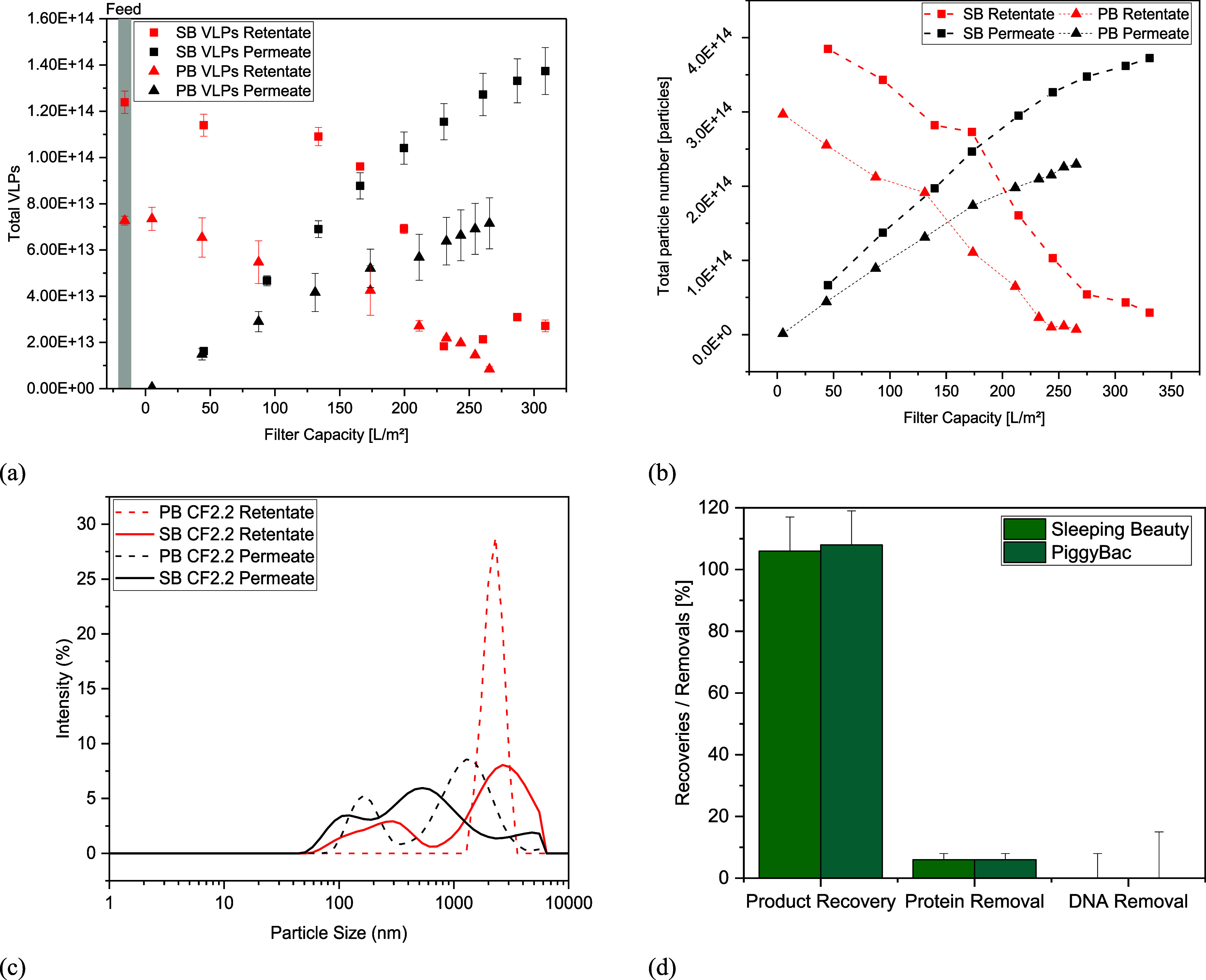
Comparison of (a) p24-ELISA, (b) NTA, (c) DLS data, and (d) VLP recoveries and protein and DNA removals for both TFDF runs.

Comparison of DLS data from the permeate stream (Figure 5c) with the cultivation supernatant (Figure 2g) shows an increase of particles in the range of 400 nm to 2 μm in the permeate. The permeate from PB contained 2.7 times more particles in the size range of 1–3 μm, which probably passed through first and were increasingly separated in the filter as the harvest progressed.

Both harvests can be well described by the standard mechanism calculated from the first concentration step, meaning the deposition of the particles within the pores, whereby the blocking constant for PB at 0.23 is slightly higher than that for SB at 0.16. A higher blocking constant indicates a faster decrease in the LMH (Figure 4b). However, the harvest of the SB cultivation can also be described very well, with the blocking constant of the PB harvest up to a filter capacity of 80 L/m^2^. Consequently, a slower deposition of SB-generated particles within the pores can be observed only from a filter capacity of 80 L/m^2^.

During the entire harvest, no decrease in product permeability due to blocking of the membrane can be seen in either test (Figure 5a). The DLS measurements (Figure 5c) show that, as expected, only a small proportion of particles in the permeate are >2 μm. At the same time, the main peak of the retentate for both harvests is >1 μm. In the harvest of the SB cultivation, there are also particles in the size range of the product in the retentate, which, does not, however, have any influence on the recovery achieved.

As a result, the minimum recovery value is 96% for PB and 95% for SB. This is in line with the literature, which documents yields of 88–100% for harvesting lentiviral vectors using TFDF.^19,20^ Due to the cutoff of 2–5 μm, a reduction of the main contaminants, DNA and proteins, is not to be expected and is therefore in the lower single-digit range (Figure 5d). NTA measurements show a decrease in the retentate and simultaneous accumulation of particles in the measurable range over the course of filtration (Figure 5b). Where for the SB run, the total number of particles found in the permeate is 97%, only 72% of the total particles are found at the end of filtration for TFDF harvest with PB. Nonetheless, no loss in product recovery could be observed despite a significant loss of particles tracked by NTA. A possible explanation could be that the PB harvest contained more larger particles and they accumulated in the depth filter layer of the membrane, therefore leading to a loss on the permeate site. This is backed by DLS measurement of the TFDF feed, as pellet and supernatant distributions of PB are generally skewed toward particles in the 0.75–2 μm range.

### 3.4 Ultra-/diafiltration

**Figure 6:**
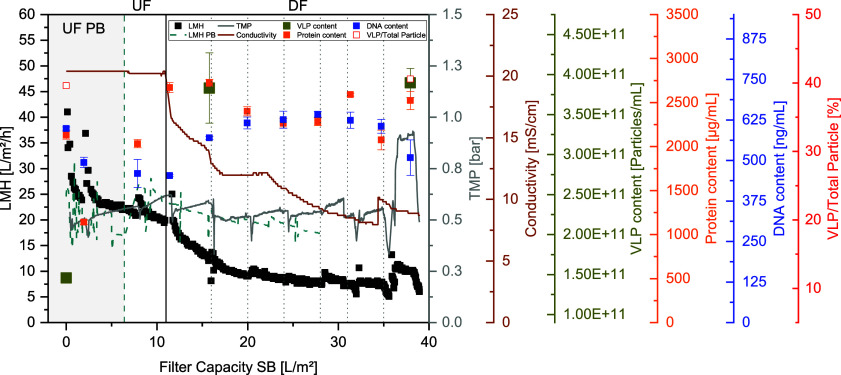
Process performance data for SB and PB as well as impurity and product data for SB filtration. Conductivity measurements were taken at-line.

After harvesting, ultrafiltration and diafiltration are carried out to concentrate the feed and condition it with the loading buffer for subsequent chromatography. The applied process parameters are displayed in Table 2. The PB-based reference run was performed with only two-thirds of the SB feed volume, and therefore, the total filter capacities vary between both runs (Figure 6). TMP courses between both runs were comparable with a set point of 0.5 bar with an apparent increase for the last DV due to trapped air in the system. A comparison of process performance during the first concentration was also not applicable as a device error during the PB run led to incorrect flux data (gray area in Figure 6). During SB UFDF, the LMH initially decreases sharply until a constant value is reached. The LMH is approximately 40 L/m^2^/h at the beginning and reaches a plateau at around 20 L/m^2^/h after the first concentration. Throughout the course of seven diafiltration volumes, the LMH for both runs decreased but with a 53% higher LMH for PB than for the SB run at the end of the process. However, since the feed from SB is significantly more concentrated in terms of product and total particle count as well as in terms of impurities, proteins, and DNA, a lower LMH is not unusual.

**Table 2:** Process Data and Comparison of Minimal LMH during UF/DF

		PB	SB
feed volume (mL)		150	240
process step 1		Conc. (VCF 3), 0.5 bar TMP	
process step 2		DF (7 DV), 0.5 bar TMP	
UF	filter capacity (L/m^2^)	6	11
DF	filter capacity (L/m^2^)	23	28

**Figure 7:**
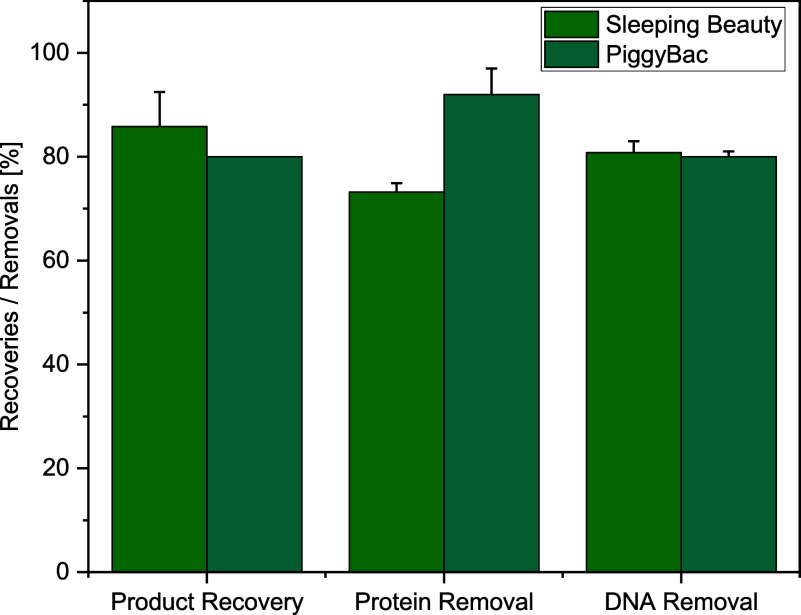
VLP recoveries and protein and DNA removals for both UFDF runs.

**Figure 8:**
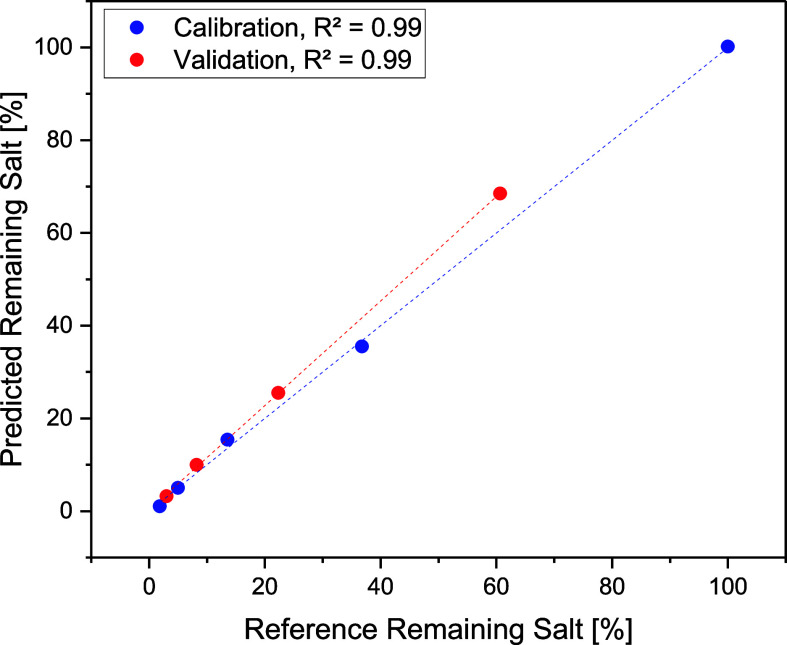
Change in the FTIR spectrum with increasing diafiltration volumes. The figure shows the fingerprint region used for further processing.

In order to ensure a robust process and the same final concentration for both cell lines with the same initial volume, a dynamic adjustment of the concentration is necessary, which can be automated by integrating the PAT strategy presented above from the USP as well as from a previously published control strategy.^6,17^ During the diafiltration, constant conductivity and FTIR measurements of the process medium can provide information about the progress of buffer exchange, which therefore can be used to optimize and control the respective exchange rate. Conductivity data of the retentate show a steady value while concentrating the feed but a change while diafiltrating the feed with the AEX loading buffer (Figure 8). As the conductivity of the loading buffer is 7.5 mS/cm, an endpoint of the diafiltration can be determined via conductivity measurement. FTIR measurements were also taken at-line with the same settings applied during cultivation. The VLP recoveries and protein and DNA removals for both UFDF runs are shown in Figure 7.

**Figure 9:**
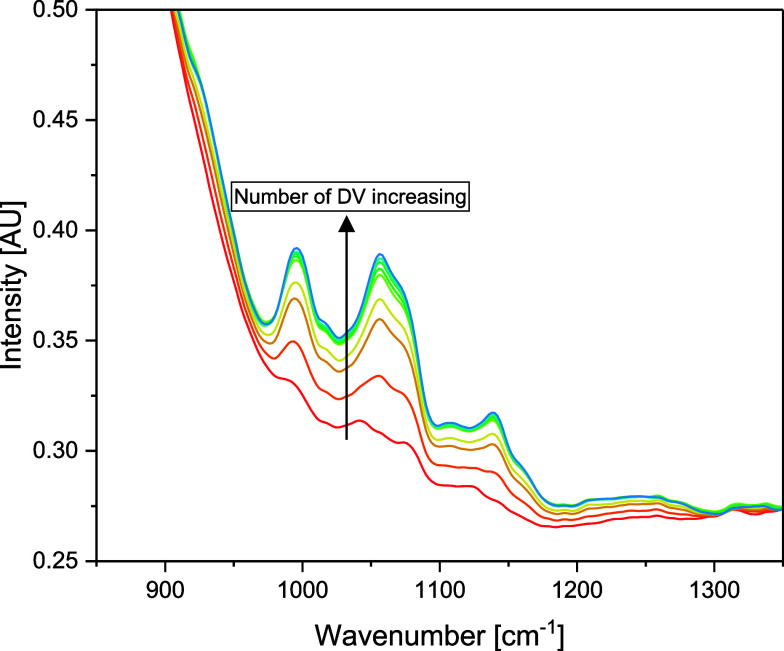
PLSR model of calibration and validation data sets.

Figure 8 shows the change in the FTIR spectrum while diafiltrating. A significant rise in peak intensity for the fingerprint region can be observed when the retentate contains a higher proportion of the diafiltrating buffer. The three main peaks can be assigned to sucrose. With a mass fraction of 5%, it is the most abundant substance in the buffer, and literature data indicate a similar FTIR spectrum for sucrose^33,34^ This change can be used to calculate the progress in buffer exchange when the measured spectrum is compared to the pure diafiltrating buffer as a function of the remaining buffer species still present in the retentate. PLSR of the spectra results in a prediction plot of the remaining salt species shown in Figure 9. With correlation coefficients of *R*^2^ = 0.99, FTIR measurements are suitable for controlling the diafiltration step. FTIR was already shown to be highly sensitive when buffer compositions change and hence used to identify impurities in a biopharmaceutical process.^35^

The lower LMH observed in SB had no influence on product recovery and removal of impurities. For both cell lines, >80% of the product could be recovered and >73% of all proteins and >80% of the DNA could be separated (see Table 3). As the feed volume is reduced during the concentration step, DNA and total protein are reduced, indicated by a decrease in contents’ concentration while VLP concentrations rise. During diafiltration, concentration values fluctuate but remain constant for the impurities and product. The VLP to total particle ratio also remained constant over the course of the process. Product permeability could not be observed over the course of filtration (data not shown), which leads to the conclusion that the lost particles were adsorbed on the membranes’ surface. This already had been observed in the literature with a membrane that had the same cut-off used in this study.^36^ Degradation of particles is not suspected as previous experiments were conducted that assessed the maximum tolerable shear rate without particle loss during filtration.^17^

**Table 3:** Recoveries and Removal Values for PB and SB AEX

	SB	PB
VLP load (particles)	6.2 × 10^10^ ± 2.35E9	5.8 × 10^10^ ± 7.6E9
DNA load (ng)	1470 ± 44	3982 ± 9
protein load (μg)	2036 ± 406	516 ± 42
product recovery (%)	94 ± 4	99 ± 12
protein removal (%)	35 ± 8	31 ± 1
DNA removal (%)	7 ± 5	3 ± 1

### 3.5 Anion-Exchange Chromatography

**Figure 10:**
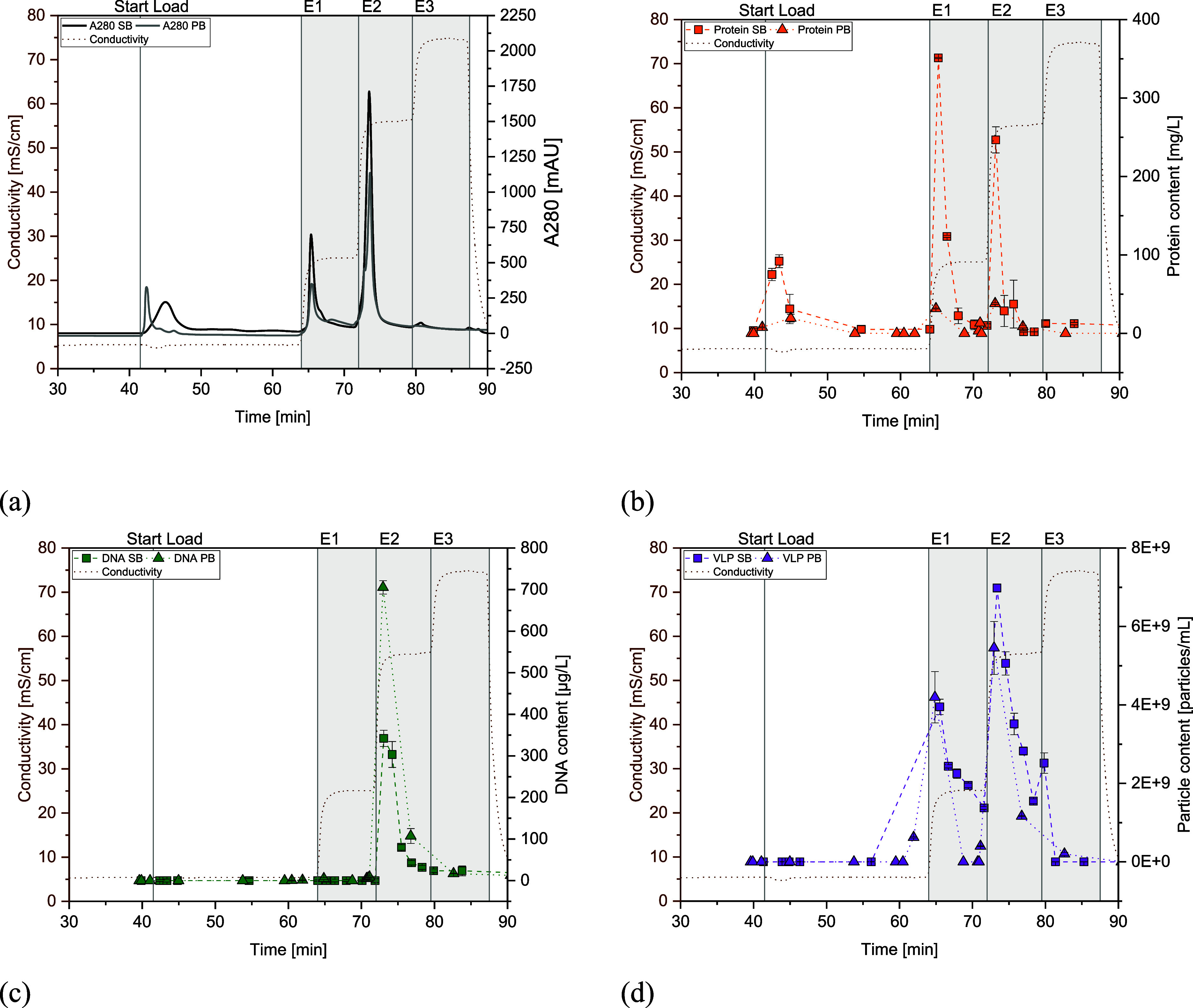
Comparison of AEX chromatograms from load to end of elution between PB and SB runs. (a) Comparison of A280 signals, (b) comparison of protein impurity data, (c) comparison of DNA impurity data, and (d) comparison of VLP product data.

After UF/DF, the product is purified by AEX, with the same method applied for both feeds to elute the product within a three-step salt gradient. To compare the performance of this step, the same amount of product was loaded onto the column for each feed with a lower amount of protein and a higher DNA content for SB loaded onto the column compared to the PB run. The total product load is 80% of the maximum capacity determined by DBC (data not shown). Figure 10a shows comparable absorption signals at 280 nm. Both impurities and product eluted comparably for both cell lines, with a higher proportion of VLPs eluting in the second elution step (E2, Figure 10b). The differences in product concentration for each fraction result from minor adaptions made for the fraction size. Data from p24-ELISA suggest an elution of the product in both fractions E1 and E2. Those findings were also described in the literature, and it is assumed that different populations within the product pool cause this effect.^2^ The peak heights for DNA differ as expected, with a peak height for SB that is about half that of the PB run. This is consistent with the total amount of DNA loaded onto the column for each cell line (Table 3). DNA elutes completely in the second elution step, which emphasizes the strong binding of dsDNA to the resin.

Protein elution behavior is also comparable, where around 70% is recovered in elution fractions E1 and E2 with generally higher peaks for the SB run, as the protein load is 4x higher. A higher proportion of protein elutes in E1. Due to the co-elution of impurities and product, optimization could be performed for better impurity removal and to further investigate both product fractions regarding their particle integrity.

## 4 Discussion and Conclusions

In this study, HEK293 cell lines modified with different transposon constructs were used for the production and purification of Gag-VLPs to evaluate a potential platform process for its capacity to purify these different feed streams. During fed-batch cultivation, growth rate, glucose consumption, and lactate accumulation were comparable, suggesting that differences between the cell lines cannot be distinguished by monitoring the main metabolites. On the other hand, the impurity and product profile between SB and PB differed, as the SB batch had an overall higher titer and total protein content but a lower DNA content compared to the PB batch. With an overall higher productivity factor of 3.6 for SB, the cell line was more efficient in producing VLPs.

To measure VCD, metabolites, and impurities during cultivation in real time, different sensors were evaluated. For VCD, the ISM showed good agreement for predicting the measurements taken with the standard CEDEX method by quantifying only the taken images with a Laplace-like transformation feature. Error between measurements is between 3 and 10%, which is comparable to or even better than the standard CEDEX counter used in this study. The deviations are in good agreement with those in the literature.^37^

The spectroscopic sensors showed sufficient accuracy to predict the main metabolites and impurities during cultivation with SB. Correlation coefficients of 0.97 for glucose and protein with FTIR, 0.94 for DNA, and 0.81 for VLPs with DAD could be obtained. With a deviation of 0.38 g/L for glucose, the predicted model showed compliant values to the literature.^38^ The models, however, lacked the ability to identify lactate and critical amino acids like glutamine, which would improve the predictive power of a DT (data not shown). The applied PAT strategy stated in Hengelbrock et al. should undergo feasibility studies with the models developed in this study.^6^ PAT measurements in combination with predictive USP models as DTs can be combined as soft sensors for challenging species.

Harvesting of the fed-batches with a TFDF membrane in the TFF mode showed neither blocking during filtration nor loss of product permeability, demonstrating the membranes’ ability to successfully separate cells from the culture medium. Additionally, the removal of cell debris and other particles in the range of 0.75–2 μm was shown when harvesting the PB culture, as NTA measurements indicate particle loss of 72% in the permeate while recovering >95% of the VLP particles.^20^

Following the harvest, a concentration and buffer exchange by UF/DF was applied and showed comparable results for both cell lines. Further strategies for controlling the final product concentration and monitoring impurities can be achieved with the PAT models applied during the USP, while the buffer exchange rate was successfully monitored by FTIR. While the AEX step showed similar results regarding product recovery and removal of impurities, optimization can improve the removal capacities for protein and DNA impurities. Other groups showed similar results when implementing a stepwise AEX purification step.^2,24,39^ Recovering VLPs only from E1 would lead to lower recoveries (37% for SB, 41% for PB) despite the significantly better removal of total protein (60% for SB, 69% for PB) and DNA (100.0% for SB, 99.3% for PB).

In conclusion, the established process showed robust purification of Gag-VLPs with different systems applied for the production of those particles. The SB transposon vector system-based producer cells yielded a higher VLP titer, which enhanced overall productivity by a factor of 3.6 as the investigated process units did not undergo adaptions in the operating space. This emphasizes the validity of the implemented units as a platform process. In the context of PAT, the results indicate the successful usability of control strategies stated in previous publications. With modern PAT sensors such as the in situ microscope, which has also proven reliable in predicting VCD, this collectively paves the way for the development of a flexible and autonomous production platform.
